# The impact of BCG vaccination on tuberculin skin test responses in children is age dependent: evidence to be considered when screening children for tuberculosis infection

**DOI:** 10.1136/thoraxjnl-2015-207687

**Published:** 2016-06-22

**Authors:** James A Seddon, James Paton, Zohreh Nademi, Denis Keane, Bhanu Williams, Amanda Williams, Steven B Welch, Sue Liebeschutz, Anna Riddell, Jolanta Bernatoniene, Sanjay Patel, Nuria Martinez-Alier, Paddy McMaster, Beate Kampmann

**Affiliations:** 1 Department of Academic Paediatrics, Centre of International Child Health, Imperial College London, London, UK; 2 School of Medicine, College of Medical, Veterinary, and Life Sciences, University of Glasgow, Glasgow, UK; 3 Department of Paediatrics, Great North Children's Hospital, Newcastle upon Tyne, Tyne and Wear, UK; 4 Institute of Cellular Medicine, Newcastle University, Newcastle upon Tyne, Tyne and Wear, UK; 5 Department of Paediatrics, London North West Healthcare NHS Trust, Harrow, Middlesex, UK; 6 Birmingham Chest Clinic, Heart of England NHS Foundation Trust, Birmingham, West Midlands, UK; 7 Department of Paediatrics, Newham University Hospital, Barts Health NHS Trust, London, UK; 8 The Children's Hospital at the Royal London Hospital, Barts Health NHS Trust, London, UK; 9 Department of Paediatric Infectious Diseases, Bristol Royal Hospital for Children, Bristol, UK; 10 Department of Paediatric Infectious Diseases and Immunology, Southampton Children's Hospital, Southampton, UK; 11 Department of Paediatric Infectious Diseases, Evelina Children's Hospital, London, UK; 12 Department of Paediatric Infectious Diseases, North Manchester General Hospital, Manchester, UK; 13 Vaccines & Immunity Theme, Medical Research Council Unit, Atlantic Boulevard, Fajara, The Gambia

**Keywords:** Tuberculosis, Paediatric Lung Disaese, Respiratory Infection

## Abstract

**Background:**

Following exposure to TB, contacts are screened to target preventive treatment at those at high risk of developing TB. The UK has recently revised its recommendations for screening and now advises a 5 mm tuberculin skin test (TST) cut-off irrespective of age or BCG status. We sought to evaluate the impact of BCG on TST responses in UK children exposed to TB and the performance of different TST cut-offs to predict interferon γ release assay (IGRA) positivity.

**Methods:**

Children <15 years old were recruited from 11 sites in the UK between January 2011 and December 2014 if exposed in their home to a source case with sputum smear or culture positive TB. Demographic details were collected and TST and IGRA undertaken. The impact of BCG vaccination on TST positivity was evaluated in IGRA-negative children, as was the performance of different TST cut-offs to predict IGRA positivity.

**Results:**

Of 422 children recruited (median age 69 months; IQR: 32–113 months), 300 (71%) had been vaccinated with BCG. BCG vaccination affected the TST response in IGRA-negative children less than 5 years old but not in older children. A 5 mm TST cut-off demonstrated good sensitivity and specificity in BCG-unvaccinated children, and an excellent negative predictive value but was associated with low specificity (62.7%; 95% CI 56.1% to 69.0%) in BCG-vaccinated children. For BCG-vaccinated children, a 10 mm cut-off provided a high negative predictive value (97.7%; 95% CI 94.2% to 99.4%) with the positive predictive value increasing with increasing age of the child.

**Discussion:**

BCG vaccination had little impact on TST size in children over 5 years of age. The revised TST cut-off recommended in the recent revision to the UK TB guidelines demonstrates good sensitivity but is associated with impaired specificity in BCG-vaccinated children.

**Key messages**

**What is the key question?**

- The impact of BCG vaccination on tuberculin skin test response is poorly understood in children; we set out to determine the impact of previous BCG vaccination on tuberculin skin test response in UK children following household exposure to an infectious case of TB.

**What is the bottom line?**

- The effect of infant BCG vaccination on the size of the tuberculin skin test wanes as children get older.

**Why read on?**

- The revised UK national guidance of using a 5 mm tuberculin skin test cut-off results in impaired specificity in BCG-vaccinated children.

## Background

Following household exposure to an infectious adult with pulmonary TB, up to half of all children living with this source case will themselves have evidence of sensitisation to *Mycobacterium tuberculosis*. This is usually manifest as a positive immune test in either the skin or blood.^1^ TB infection implies that organisms are dormant within the body and are not causing disease; the child is clinically well with a normal chest radiograph. In the majority of children, disease never develops but over time, in a proportion, the mycobacteria will overcome the immune-mediated containment and the child will become ill with TB disease.^2^ ^3^ Certain risk factors increase the likelihood of progressing from asymptomatic TB infection to TB disease. These include young age and suppression of the immune system.^4^ ^5^ Over 50 years ago it was demonstrated that by providing drug therapy to individuals with evidence of TB infection, the risk of progression to TB disease could be significantly reduced.^6^ The use of daily isoniazid, given for at least 6 months, has subsequently been demonstrated in a large number of studies to be effective in reducing the risk of disease progression in those with TB infection for HIV-positive^7^ and HIV-negative contacts.^8^

The organism *M. tuberculosis* is not detectable in individuals with TB infection. Currently available diagnostic tests rely on demonstrating evidence of the presence (or absence) of prior immunological sensitisation. Traditionally, the tuberculin skin test (TST) has been used for this purpose. In the TST, a crude mixture of mycobacterial antigens is injected into the dermis of the skin in the forearm and the presence of a skin reaction at the site of injection ‘read’ at 48 h for evidence of a type IV delayed hypersensitivity reaction, signifying immunological memory to antigens within the tuberculin. These antigens, however, are not unique to *M. tuberculosis*; some are also found in BCG and environmental, non-tuberculous mycobacteria.^9^ Newer tests, such as the interferon γ release assays (IGRAs), measure either the quantity of interferon γ released by T cells or the number of T cells which release interferon γ, after stimulation by *M. tuberculosis*-specific antigens. Consequently, they have been found to have superior specificity.^10^

Until 2005, it was advised that all children in the UK be given BCG between the ages of 10 and 14 years. In addition, targeted vaccination, to be given soon after birth, was advised for children deemed at increased risk of TB exposure. This included children living in areas of high TB prevalence and those born within families from high incidence countries.^11^ From 2005, universal BCG vaccination was no longer recommended; targeted neonatal vaccination continued. In 2006 the National Institute for Health and Care Excellence (NICE) published guidance on the management of TB contacts.^12^ NICE suggested a stepwise screening strategy for TB infection in childhood contacts, using combinations of TST and IGRA with different algorithms for children of different ages, resulting in significant complexities in management. The 2006 NICE guidance also suggested using different TST cut-offs for children who had previously been vaccinated with BCG (15 mm induration) and those who had not been (6 mm induration). The rationale for this was that a prior BCG immunisation might lead to false-positive results and impaired specificity of the TST. However, it was felt that the size of the induration would differentiate between BCG and TB infection—the larger the induration, the greater the probability that the response was due to TB infection.^13^ It may be that part of this recommendation is a legacy from a time when BCG was given to adolescents, where BCG would have a significant impact on TST response. Many clinicians were, however, concerned that a response of less than 15 mm in a BCG-vaccinated child might still indicate TB infection, and should not be ignored. International guidelines recommend a cut-off of 10 mm induration for a positive TST result regardless of BCG status.^14^ In early 2016, NICE updated its guidance and suggested that a TST cut-off of 5 mm be used irrespective of BCG vaccination status in all ages of children.^15^

Prior to the advent of IGRA tests, it was not possible to evaluate the impact of a previous BCG vaccination on the TST response in TB contacts as it was not possible to determine whether a TST response was due to the prior BCG vaccination rather than TB infection. However, by evaluating TST responses in child contacts of TB cases shown to be IGRA-negative, the contribution of BCG can now be more precisely documented. While fully acknowledging that no gold standard for TB infection exists, we felt that a negative IGRA test result would act as a proxy measure for lack of sensitisation to *M. tuberculosis*. We therefore set out to answer two questions. First, to determine the influence of prior BCG vaccination on TST responses in IGRA-negative children living in the UK who were being screened because of household exposure to an infectious TB case. Second, to evaluate the sensitivity, specificity, positive predictive value (PPV) and negative predictive value (NPV) of different TST cut-offs to predict IGRA positivity in BCG vaccinated and unvaccinated children of different ages.

## Methods

### Study setting

This investigation was carried out within a larger study, the NIHR-funded IGRA Kids Study (NIKS), which sought to evaluate the NPV of IGRA to predict incident TB disease in children exposed to TB in the UK. Sites included five paediatric TB clinics in London, together with paediatric TB clinics in Southampton, Bristol, Birmingham, Manchester, Glasgow and Newcastle. All children (<15 years) presenting to one of the 11 participating centres between 1 January 2011 and 31 December 2014 were eligible for recruitment if they had a history of household exposure to a source case with pulmonary sputum smear or culture positive TB.

### Study procedures and definitions

Following the diagnosis of a source case of infectious pulmonary TB, household contacts were identified and screened. Screening aimed to identify contacts already unwell with TB disease and asymptomatic individuals eligible for treatment of TB infection. Families were invited to participate in the study and written informed consent was obtained. Screening for TB disease in children included history, examination, chest radiography, TST and IGRA tests, and microbiology if indicated. The evaluation of contacts to decide on the provision of treatment for TB infection was based on NICE guidelines, in conjunction with clinical context. Children with TB infection were treated with isoniazid and rifampicin daily for 3 months.

TST and IGRA were evaluated at baseline. TST was placed by experienced members of the local TB nursing teams, by injecting two tuberculin units intradermally (purified protein derivative RT23, Statens Serum Institute) with results read at 48–72 h. IGRA tests (either QuantiFERON-TB Gold In-Tube (Cellestis Ltd) or T-SPOT.TB (Oxford Immunotec Ltd) depending on the practice of the recruiting clinic) were carried out by the clinically available laboratory services, following the manufacturer's specifications. The IGRA test was repeated in all children after 2 months. If the TST was negative at baseline, it was also repeated after 2 months. TST was defined as being positive if the transverse diameter of the induration was ≥6 mm in BCG-unvaccinated children and ≥15 mm in vaccinated children, in line with the 2006 NICE recommendations. The largest TST measurement was used for analysis. The child was classified as IGRA positive (and hence assumed to be TB infected for the purposes of this analysis) if either the baseline or the 2-month IGRA was positive. Repeatedly indeterminate results were considered negative. Children were examined for the presence of a BCG scar; if present, children were classified as BCG vaccinated. If no scar was seen, but there was clear documentation in paper or electronic records, or if the parents gave a clear history of vaccination, the child was classified as BCG vaccinated. Otherwise the child was classified as BCG unvaccinated.

### Statistical analysis

Data were entered into a tailor-made online database in real time and later checked centrally for entry errors. Data were analysed using STATA software (V.11; Stata Corp, College Station, Texas, USA). For analysis, children were divided into four age groups: <2 years, 2 to <5 years, 5 to <10 years and 10 to <15 years. The Mann–Whitney test was used to assess differences between TST measurements in BCG vaccinated and unvaccinated children due to the non-normal distribution of the data. ORs and 95% CIs were calculated to determine the impact of BCG vaccination on TST positivity, in IGRA-negative children, using three TST cut-offs (5, 10 and 15 mm). Sensitivity, specificity, PPV, NPV, together with receiver operator characteristic (ROC) curves and their respective area under the curves were determined for different TST cut-offs to predict IGRA positivity. 95% CIs were determined for each of the above.

## Results

Of 422 children recruited, 216 (51%) were boys; median age was 69 months (IQR: 32–113). Of 370 children tested for HIV, none tested positive and no children in the study were known to be HIV positive. The majority of children (361; 86%) were born in the UK. Three hundred (71%) had been vaccinated with BCG (table 1).

**Table 1:** Demographic and clinical characteristics of children in the study

	IGRA status		BCG status		
Characteristic	IGRA negative (%)	IGRA positive (%)	BCG unvaccinated (%)	BCG vaccinated (%)	Total (%)
Total	314 (100)	108 (100)	122 (100)	300 (100)	422 (100)
Gender					
Male	164 (52.2)	52 (48.2)	64 (52.5)	152 (50.7)	216 (51.2)
Female	150 (47.8)	56 (51.9)	58 (47.5)	148 (49.3)	206 (48.8)
Median age in months (IQR)	60 (25–104)	94 (61–136)	71 (37–118)	68 (30–110)	69 (32–113)
Ethnicity					
White	69 (22.0)	36 (33.3)	84 (68.9)	21 (7.0)	105 (24.9)
Indian	38 (12.1)	10 (9.3)	3 (2.5)	45 (15.0)	48 (11.4)
Pakistani	60 (19.1)	18 (16.7)	8 (6.6)	70 (23.3)	78 (18.5)
Bangladeshi	34 (10.8)	4 (3.7)	2 (1.6)	36 (12.0)	38 (9.0)
Black Caribbean	2 (0.6)	0	0	2 (0.7)	2 (0.5)
Black African	68 (21.7)	27 (25.0)	12 (9.8)	83 (27.7)	95 (22.5)
Black Other	8 (2.6)	6 (5.6)	5 (4.1)	9 (3.0)	14 (3.3)
Chinese	1 (0.3)	0	0	1 (0.3)	1 (0.2)
Mixed/other	34 (10.8)	7 (6.5)	8 (6.6)	33 (11.0)	41 (9.7)
HIV status*					
Negative	280 (89.2)	90 (83.3)	100 (82.0)	270 (90)	370 (87.7)
Positive	0	0	0	0	0
Not tested	34 (10.8)	18 (16.7)	22 (18.0)	30 (10)	52 (12.3)
Born in the UK					
No	36 (11.5)	25 (23.2)	12 (9.8)	49 (16.3)	61 (14.5)
Yes	278 (88.5)	83 (76.9)	110 (90.2)	251 (83.7)	361 (85.6)
Type of IGRA					
QuantiFERON-TB Gold	190 (60.7)	71 (65.7)	84 (68.9)	177 (59.2)	261 (62.0)
T-SPOT.TB	124 (39.3)	37 (34.3)	38 (31.2)	122 (40.8)	161 (38.0)

*HIV status was known in 370 children. No children were known to be HIV positive.

IGRA, interferon γ release assay.

In IGRA-negative children <2 years, there was a difference in TST size between those who were BCG vaccinated and those who were unvaccinated (median: 4 mm (IQR: 0–12) vs median: 0 mm (IQR: 0–0); p<0.001; table 2). There was also a difference for children aged 2 years to <5 years (p=0.001) but no significant difference was seen in children aged 5 to <10 years (p=0.12) or children aged 10 to <15 years (p=0.09) (table 2). For IGRA-negative children aged <2 years and 2 to <5 years, those who were BCG vaccinated were more likely to have a positive TST result than those who were unvaccinated, at all three TST cut-offs. For children aged 5 to <10 years and 10 to <15 years there was a smaller and less significant difference in TST positivity between children who were BCG vaccinated and those who were unvaccinated, at all TST cut-offs (table 3).

**Table 2:** Median TST induration in BCG-vaccinated and BCG-unvaccinated children at different ages who are IGRA negative

	All children		BCG vaccinated		BCG unvaccinated		
Age (years)	Number	Median TST (IQR)	Number	Median TST (IQR)	Number	Median TST (IQR)	p Value
All children	314	0 (0–6)	228	0 (0–10)	86	0 (0–0)	<0.001
0 to <2	76	0 (0–10)	57	4 (0–12)	19	0 (0–0)	<0.001
2 to <5	80	0 (0–4)	55	0 (0–8)	25	0 (0–0)	0.001
5 to <10	102	0 (0–5)	75	0 (0–10)	27	0 (0–3)	0.12
10 to <15	56	0 (0–7)	41	0 (0–8)	15	0 (0–0)	0.09

IGRA, interferon γ release assay; TST, tuberculin skin test.

**Table 3:** Odds of having a positive TST response of 5 mm, 10 mm and 15 mm between IGRA negative children who had been vaccinated with BCG at birth versus children who had not been vaccinated with BCG

	5 mm		10 mm		15 mm	
Age (years)	OR (95% CI)	p Value	OR (95% CI)	p Value	OR (95% CI)*	p Value
All children	5.80 (2.58 to 13.0)	<0.001	4.03 (1.73 to 9.39)	<0.001	3.04 (1.14 to 8.10)	0.02
0 to <2	16.2 (1.69 to 155)	0.001	8.31 (0.94 to 73.5)	0.02	−	0.05
2 to <5	9.85 (1.11 to 87.7)	0.01	7.43 (0.85 to 65.1)	0.03	6.00 (0.69 to 52.4)	0.06
5 to <10	3.05 (0.93 to 10.0)	0.05	2.71 (0.72 to 10.3)	0.13	1.92 (0.39 to 9.52)	0.41
10 to <15	4.16 (0.77 to 22.3)	0.07	2.10 (0.39 to 11.2)	0.38	0.90 (0.15 to 5.32)	0.91

*Unable to calculate ORs where a zero exists in one of the four cells needed to generate the OR. No BCG-unvaccinated children under 2 years with negative IGRA results had a TST induration of greater than 15 mm. IGRA, interferon γ release assay; TST, tuberculin skin test.

**Figure 1:**
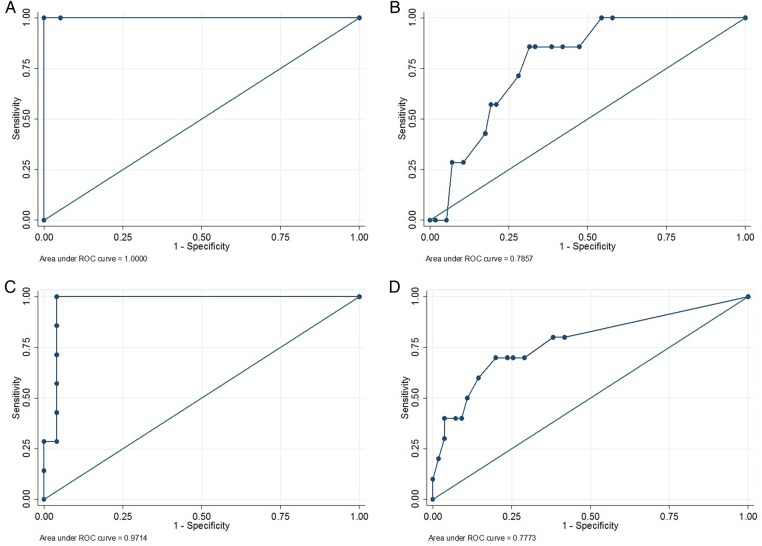

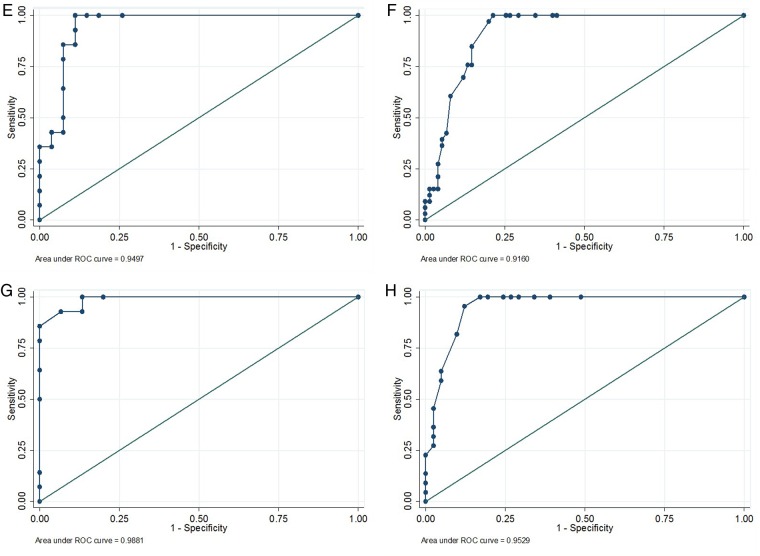
Receiver operating characteristic curves for tuberculin skin test results of different cut-offs to predict interferon γ release assay positivity. (A) BCG-unvaccinated children <2 years (n=20; AUC=1.00; 95% CI not calculable). (B) BCG-vaccinated children <2 years (n=64; AUC=0.79; 95% CI 0.65 to 0.93). (C) BCG-unvaccinated children aged 2 to <5 years (n=32; AUC=0.97; 95% CI 0.91 to 1.00). (D) BCG-vaccinated children aged 2 to <5 years (n=65; AUC=0.78; 95% CI 0.60 to 0.95). (E) BCG-unvaccinated children aged 5 to <10 years (n=41; AUC=0.95; 95% CI 0.88 to 1.00). (F) BCG-vaccinated children aged 5 to <10 (n=108; AUC=0.92; 95% CI 0.86 to 0.97). (G) BCG-unvaccinated children aged 10 to <15 (n=29; AUC=0.99; 95% CI 0.96 to 1.00). (H) BCG-vaccinated children aged 10 to <15 (n=63; AUC=0.95; 95% CI 0.90 to 1.00).

In BCG-unvaccinated children, the use of a 5 mm or a 10 mm TST cut-off to predict IGRA positivity resulted in similar sensitivities and specificities and provided excellent NPVs at all ages (table 4). For all ages of unvaccinated children, the area under the ROC curve was above 0.94 (figure 1). For children under 5 years who had been vaccinated with BCG, the sensitivity and specificity of TST to predict IGRA positivity was low using all three cut-offs (table 5). In older children, good sensitivities were seen using 5 or 10 mm, but at 5 mm specificity was poor. By using a 15 mm cut-off the specificity improved, but at the expense of sensitivity. For BCG-vaccinated children under 5 years, the area under the ROC curve was below 0.8. However, in children over 5 years, the area under the curve was greater than 0.9.

**Table 4:** Sensitivity, specificity, PPVs and NPVs for different TST cut-offs in correctly identifying IGRA positivity in BCG-unvaccinated children

	5 mm (with 95% CIs)				10 mm (with 95% CIs)				15 mm (with 95% CIs)			
Age (years)	Sensitivity	Specificity	PPV	NPV	Sensitivity	Specificity	PPV	NPV	Sensitivity	Specificity	PPV	NPV
All children	100 (90.3 to 100)	90.7 (82.5 to 95.9)	81.8 (67.3 to 91.8)	100 (95.4 to 100)	97.2 (85.5 to 99.9)	91.9 (84.0 to 96.7)	83.3 (68.6 to 93.0)	98.8 (93.2 to 100)	86.1 (70.5 to 95.3)	94.2 (87.0 to 98.1)	86.1 (70.5 to 95.3)	94.2 (87.0 to 98.1)
0 to <2	100 (2.5 to 100)	94.7 (74.0 to 99.9)	50.0 (1.3 to 98.7)	100 (81.5 to 100)	100 (2.5 to 100)	94.7 (74.0 to 99.9)	50.0 (1.3 to 98.7)	100 (81.5 to 100)	100 (2.5 to 100)	100 (82.4 to 100)	100 (2.5 to 100)	100 (82.4 to 100)
2 to <5	100 (59.0 to 100)	96.0 (79.7 to 99.0)	87.5 (47.4 to 99.7)	100 (85.8 to 100)	85.7 (42.1 to 99.6)	96.0 (79.7 to 99.9)	85.7 (42.1 to 99.6)	96.0 (79.7 to 99.9)	71.4 (29.0 to 96.3)	96.0 (79.7 to 99.9)	83.3 (35.9 to 99.6)	92.3 (74.9 to 99.0)
5 to <10	100 (76.8 to 100)	85.2 (66.3 to 95.8)	77.8 (52.4 to 93.6)	100 (85.2 to 100)	100 (76.8 to 100)	88.9 (70.8 to 97.7)	82.4 (56.6 to 96.2)	100 (85.8 to 100)	85.7 (57.2 to 98.2)	92.6 (75.7 to 99.1)	85.7 (57.2 to 98.2)	92.6 (75.7 to 99.1)
10 to <15	100 (76.8 to 100)	86.7 (59.5 to 98.3)	87.5 (61.7 to 98.5)	100 (75.3 to 100)	100 (76.8 to 100)	86.7 (59.5 to 98.3)	87.5 (61.7 to 98.5)	100 (75.3 to 100)	92.9 (66.1 to 99.8)	86.7 (59.5 to 98.3)	86.7 (59.5 to 98.3)	92.9 (66.1 to 99.8)

IGRA, interferon γ release assay; NPV, negative predictive value; PPV, positive predictive value; TST, tuberculin skin test.

**Table 5:** Sensitivity, specificity, PPVs and NPVs for different TST cut-offs in correctly identifying IGRA positivity in BCG-vaccinated children

	5 mm (with 95% CIs)				10 mm (with 95% CIs)				15 mm (with 95% CIs)			
Age (years)	Sensitivity	Specificity	PPV	NPV	Sensitivity	Specificity	PPV	NPV	Sensitivity	Specificity	PPV	NPV
All children	94.4 (86.4 to 98.5)	62.7 (56.1 to 69.0)	44.4 (36.4 to 52.7)	97.3 (93.2 to 99.3)	94.4 (86.4 to 98.5)	73.7 (67.5 to 79.3)	53.1 (44.1 to 62.0)	97.7 (94.2 to 99.4)	77.8 (66.4 to 86.7)	84.2 (78.8 to 88.7)	60.9 (50.1 to 70.9)	92.3 (87.8 to 95.5)
0 to <2	85.7 (42.1 to 99.6)	52.6 (39.0 to 66.0)	18.2 (7.0 to 35.5)	96.8 (83.3 to 99.9)	85.7 (42.1 to 99.6)	68.4 (54.8 to 80.1)	25.0 (9.8 to 46.7)	97.5 (86.8 to 99.9)	42.9 (9.9 to 81.6)	82.5 (70.1 to 91.3)	23.1 (5.0 to 53.8)	92.2 (81.1 to 97.8)
2 to <5	70.0 (34.8 to 93.3)	70.9 (57.1 to 82.4)	30.4 (13.2 to 52.9)	92.9 (80.5 to 98.5)	70.0 (34.8 to 93.3)	76.4 (63.0 to 86.8)	35.0 (15.4 to 59.2)	93.3 (81.7 to 98.6)	70.0 (34.8 to 93.3)	80.0 (67.0 to 89.6)	38.9 (17.3 to 64.3)	93.6 (82.5 to 98.7)
5 to <10	100 (89.4 to 100)	65.3 (53.5 to 76.0)	55.9 (42.4–68.8)	100 (92.8 to 100)	100 (89.4 to 100)	74.7 (63.3 to 84.0)	63.5 (49.0 to 76.4)	100 (93.6 to 100)	75.8 (57.7 to 88.9)	86.7 (76.8 to 93.4)	71.4 (53.7 to 85.4)	89.0 (79.5 to 95.2)
10 to <15	100 (84.6 to 100)	61.0 (44.5 to 75.8)	57.9 (40.8–73.7)	100 (86.3 to 100)	100 (84.6 to 100)	75.6 (59.7 to 87.6)	68.8 (50.0 to 83.9)	100 (88.8100)	95.5 (77.2 to 99.9)	87.8 (73.8 to 95.9)	80.8 (60.7 to 93.5)	97.3 (85.8 to 99.9)

IGRA, interferon γ release assay; NPV, negative predictive value; PPV, positive predictive value; TST, tuberculin skin test.

## Discussion

This large UK cohort study of paediatric household TB contacts shows that BCG vaccination had little effect on TST size in children over 5 years of age. In younger children, BCG did affect TST measurements, but as age increased this effect was less pronounced. In BCG-unvaccinated children, 5 and 10 mm TST cut-offs showed good sensitivity and specificity to detect TB infection, defined as IGRA positivity, while 15 mm was associated with impaired sensitivity. In BCG-vaccinated children, TST performed poorly in children under 5 years in terms of the sensitivity and specificity to predict IGRA positivity, but appeared to be reliable in children over this age, with 10 and 15 mm cut-offs providing reasonable sensitivity and specificity.

Although this topic has been evaluated in other settings, we have not been able to identify any studies carried out previously in the UK. A systematic review of studies that evaluated the impact of BCG on TST found that if vaccination was given at birth, the impact on TST size waned fairly rapidly and by the age of 10 years less than 1% of children had a TST of 10 mm or greater.^16^ If BCG was given after the first year of life, however, a much higher proportion of children remained TST positive. In line with our own data, a recent study from Spain found that the risk of a false-positive TST result, due to BCG given at birth, disappeared by 3 years post vaccination.^17^ Similar findings were also reported from a study conducted in Canada, where the data showed that when using a 5 mm cut-off, TST positivity was higher in children of all ages who had been vaccinated at birth compared with unvaccinated children. However, after 1 year, when using a cut-off of 15 mm, the rate of TST positivity was no different in vaccinated children. When using a cut-off of 10 mm, more vaccinated children had a positive TST at the age of 1 year, but by 4 years of age, no differences were seen.^18^ Using different TST size cut-offs for BCG vaccinated and unvaccinated children is not a universal policy. A recent review of national and international childhood TB guidelines found that most countries and agencies use a 10 mm induration cut-off for all children irrespective of BCG vaccination status,^19^ in line with the advice of WHO.^14^

To inform policy it is important to obtain evidence from the population for whom the policy is intended. To this end, this large study of children living in the UK, evaluated post exposure to infectious cases of pulmonary TB, is informative. This is especially so in the context of the recent revision to the NICE guidelines for the management of TB in the UK where a TST cut-off of 5 mm has been recommended for all ages of children irrespective of BCG vaccination history.^15^

This study has some acknowledged limitations. The first limitation was that we used the UK NICE 2006 definitions of TST positivity to decide if a second TST was required. Therefore, for BCG-unvaccinated children, those with an initial TST of ≥6 mm did not have a repeat test after 2 months. Although this pragmatic approach reflected UK policy, it may have resulted in TST measurements for BCG-vaccinated children being lower than if we had repeated the TST in all children and used the largest TST response in analysis. A second limitation was that as this study was carried out under programmatic conditions, some data were missing. Although, the absolute numbers were small, the most common missing data were repeat TST testing for those children with initially negative tests (five children). Third, as we were using routine, clinical data for IGRA testing, some centres used QuantiFERON-TB Gold In-Tube and some T-SPOT.TB. Although it would have been ideal to have used the same test for all children, we, and other research groups, have previously shown these two IGRA tests to be highly concordant and it was a deliberate decision of the principal investigator and the collaborators to use their locally available IGRA.^10^ ^20–22^ Fourth, we used evidence of a BCG scar, documentation of vaccination, or a clear history of vaccination from the parents as evidence of BCG vaccination. It is possible that some children were incorrectly classified using this approach. Fifth, although the majority of children were vaccinated soon after birth, we were unable to document exactly when each child received the BCG. This makes it more difficult to determine how quickly the effects of BCG on TST responses wane. Sixth, this study was carried out in the UK and caution should be exercised when generalising results to other contexts. We anticipate that similar findings would be seen in countries with a similar epidemiological TB pattern but it should be acknowledged that the PPVs and NPVs would likely change with the background TB prevalence and consequent pre-test probability that the child was IGRA positive. Seventh, although all children were exposed to TB in their households, it is theoretically possible that children living in areas that recommended BCG vaccination were, in some way, more exposed to TB than children from other regions in the UK. This could have led to some degree of selection bias that might have impacted on TST responses. Eighth, it is possible that in some of the age subgroups, the sample size was not large enough to detect modest variations in TST response due to BCG, especially in older children. Finally, and most importantly, we used the IGRA result as the ‘gold standard’ to define who was genuinely infected and compared different TST cut-offs in BCG vaccinated and unvaccinated children against this result. It is acknowledged that IGRA tests have imperfect sensitivity and specificity, especially in younger children.^10^ ^22^ It is therefore possible that some IGRA-negative children who had a reactive TST were actually infected and their positive TST response may have been due to *M. tuberculosis.* These would have been classified as false positives. Conversely some IGRA-positive children with unreactive TST responses may not have been infected with *M. tuberculosis*. These children would have been classified as false negatives. As we sought to determine the impact of BCG on TST responses in children, we used IGRA as a tool to screen out the majority of the TST responses that were caused by genuine TB infection and not BCG. Although there may have been one or two misclassifications, we are confident that the overall result is valid.

Our study suggests that in the youngest children BCG does affect TST measurements. However, these children are the most vulnerable to progression from infection to disease and are also the most susceptible to severe disseminated forms of TB disease, such as TB meningitis and miliary TB. Because of this, greater screening test sensitivity and a high NPV is more important than specificity and PPV for many clinicians when evaluating children in this age group. WHO recommends treating all children aged under 5 years for TB infection following significant exposure to an infectious TB case, irrespective of the result of the test of TB infection and irrespective of BCG vaccination status.^23^

For those managing TB in children, there is a need to consider the interpretation of screening tests. As no tests are perfect there will always be a trade-off between sensitivity and specificity of the test chosen as well as their respective PPVs and NPVs. The question of whether it is better to treat some children for TB infection unnecessarily (because of greater test sensitivity) or to fail to treat some children with TB infection who may progress to disease (because of greater test specificity) remains challenging. Although 6 months of daily isoniazid is a long course of treatment for a child who is well, it is safe^24^ and effective.^8^ ^25^ Three months of daily isoniazid and rifampicin has been shown to be an appropriate alternative strategy.^26^ Ideally, tests with a high NPV are used for screening, while tests for diagnosis should have a high PPV. However, both false negatives and false positives have clinical and cost implications. Modelling exercises suggest that not only is the treatment of TB infection in children likely to be a highly cost-effective strategy, but in children under 2 years old, screening for exposure and treating without testing for evidence of infection is the most cost-effective intervention.^27^ As newer, simpler treatments become available, such as the 3-month duration once-a-week regimen with rifapentine and isoniazid,^28^ ^29^ the benefits of treating may increasingly outweigh the risks and costs and of not treating younger children.

## Conclusion

Our data suggest that the impact of infant BCG vaccination on TST responses in children exposed to TB wanes with age. In BCG-vaccinated children a TST cut-off of 5 mm is associated with poor specificity.
